# Proline Modulates the *Trypanosoma cruzi* Resistance to Reactive Oxygen Species and Drugs through a Novel D, L-Proline Transporter

**DOI:** 10.1371/journal.pone.0092028

**Published:** 2014-03-17

**Authors:** Melisa Sayé, Mariana R. Miranda, Fabio di Girolamo, María de los Milagros Cámara, Claudio A. Pereira

**Affiliations:** Laboratorio de Biología Molecular de Trypanosoma cruzi (LBMTC), Instituto de Investigaciones Médicas “Alfredo Lanari”, Universidad de Buenos Aires and CONICET, Buenos Aires, Argentina; Instituto Butantan, Laboratório Especial de Toxinologia Aplicada, Brazil

## Abstract

*Trypanosoma cruzi,* the etiological agent of Chagas' disease, has a metabolism largely based on the consumption of glucose and proline. This amino acid is essential for host cells infection and intracellular differentiation. In this work we identified a proline transporter (TcAAAP069) by yeasts complementation assays and overexpression in *Trypanosoma cruzi* epimastigotes. TcAAAP069 is mono-specific for proline but presents an unusual feature; the lack of stereospecificity, because it is competitively inhibited by the D- enantiomer. Parasites overexpressing TcAAAP069 have an increased intracellular proline concentration, 2.6-fold higher than controls, as a consequence of a higher proline transport rate. Furthermore, augmented proline concentration correlates with an improved resistance to trypanocidal drugs and also to reactive oxygen species including hydrogen peroxide and nitric oxide, emulating natural physiological situations. The IC_50_s for nifurtimox, benznidazole, H_2_O_2_ and NO^.^ were 125%, 68%, 44% and 112% higher than controls, respectively. Finally, proline metabolism generates a higher concentration (48%) of ATP in TcAAAP069 parasites. Since proline participates on essential energy pathways, stress and drug resistance responses, these results provide a novel target for the development of new drugs for the treatments for Chagas' disease.

## Introduction

*Trypanosoma cruzi,* the etiological agent of Chagas' disease, has a metabolism largely based on the consumption of amino acids, mainly proline, which constitutes the main carbon and energy source in the insect stage of the parasite life cycle. L-proline is an important metabolite since it is involved in the differentiation of intracellular epimastigote to trypomastigote forms, which is required for the establishment of infection in the mammalian host [1]. Previous works showed that L-proline is taken up from the extracellular media through two active transport systems [2] and then converted into intermediates of the Krebs cycle, pyruvate, glutamate and aspartate, which are rapidly metabolized [3], [4]. Recently, the proline oxidation pathway was described in *T. cruzi*. L-proline is oxidized to pyrroline-5-carboxylate by a mitochondrial proline dehydrogenase regulating the redox state of the cell and the respiratory metabolism [5]. Proline also participates in different mechanisms of stress resistance. The ability of proline to suppress reactive oxygen species (ROS) and apoptosis in mammalian cells involves the secondary amine of its pyrrolidine ring, and it was demonstrated manipulating intracellular proline concentration by adding the amino acid to the medium or endogenously by altering proline dehydrogenase levels [6], [7]. In addition, osmotic stress that occurs within the gut of the triatomine vector and also in the vertebrate host, initially causes swelling, but this is rapidly adjusted by a compensatory volume reversal associated with a selective amino acid efflux, mainly alanine and proline [8].

Not only L-proline is present in *T. cruzi*, the D-enantiomer was also found in these parasites, even though its roles were only partially elucidated. Proline racemases interconvert the L and D-enantiomers of proline. The description of a mitogen with proline racemase activity raised the possibility for the existence of a D-amino acid metabolism in *T. cruzi* [9]. It was proposed that this enzyme might take part in a chemical compartmentalization of the intracellular pool of proline, which is a major energy reservoir for the intracellular stages differentiation. Two proline racemase isoforms are present in *T. cruzi*; they are differentially expressed during parasite development and participate in mechanisms of virulence acquisition [10]. In addition to free D-amino acids, non-infective epimastigote and infective metacyclic parasite samples possess peptides composed of D-proline probably providing resistance against host proteolytic mechanisms [11].

In *T. cruzi*, proline uptake is mediated by two different transport systems. A low affinity, high specificity, system B; and a high affinity, low specificity, system A [2]. Recently, a new neutral amino acid transporter that translocates proline and alanine was described in *Leishmania donovani*. This transporter is the unique supplier for the intracellular pool of proline and contributes to the alanine pool; it is essential for cell volume regulation after osmotic stress; and it regulates transport and homeostasis of other unrelated amino acids [12].

The effects of interfering L-proline transport and metabolism was recently studied using the proline analogue L-thiazolidine-4-carboxylic acid (T4C). Evidences suggest that T4C affects the parasites viability and also other aspects of the *T. cruzi* life cycle especially under nutrient starvation and oxidative stress conditions [13].

The first multigenic family of amino acid transporters from *T. cruzi* (TcAAAP; Amino Acid/Auxin Permeases; TC 2.A.18) was identified by our group [14]. One interesting feature of these permeases is the absence of orthologs in mammalian genomes [15]. Few members of this family have been characterized in trypanosomatids, including an arginine and lysine permeases [16], [17], [18], [19].

In this work we have identified and functionally characterized a novel proline permease from the TcAAAP family. Since this transporter has many differential features compared to those present in mammalian host cells and proline is essential for parasite survival, we hypothesize that it is possible to develop a specific inhibitor of this *T. cruzi* permease with a minimal effect on the host cells.

## Materials and Methods

### Cell cultures

Epimastigotes of Y (DTU *T. cruzi* II) and MJ Levin (DTU *T. cruzi* I) strains [16] were cultured at 28 °C in plastic flasks (25 cm^2^), containing 5 mL of LIT medium (started with 10^6^ cells per milliliter) supplemented with 10% fetal calf serum, 100 U/mL penicillin, and 100 μg/mL streptomycin [20]. The parasites were subcultured with passages each 7 days. Cells were counted using a hemocytometer.

Stress assays were performed by adding the indicated concentrations of the drugs to 1.5 mL aliquots containing 5×10^6^ parasites in LIT medium and then were cultivated during 48-96 h in 24-wells plates. After this period cells were counted using a hemocytometer.

The *S. cerevisiae* strain MG266 (gap1, put4) [21] was kindly provided by Dr. Susana Correa, FCEN-UBA, Argentina). MG266 strain was maintained on complete yeast extract/peptone/dextrose medium. Ura^+^ transformants were selected on SC medium, which is composed by 2% glucose, 0.17% yeast nitrogen base (without amino acids), 0.5% ammonium sulfate and 2% agar. Yeasts were transformed with the pDR196 or p416 vectors containing ten different TcAAAP genes and empty vectors were used as controls, according to Gietz and Woods [22].

### Proline transport assays

Aliquots of epimastigote cultures (10^7^ parasites) or yeast cultures (1 OD) were centrifuged at 8,000 × g for 30 s, and washed once with phosphate-buffered saline (PBS). Cells were resuspended in 0.1 mL PBS and then added 0.1 mL of the transport mixture containing 1 mM L-(^3^H) proline (PerkinElmer's NEN® Radiochemicals; 0.4 μCi). Following incubation at 28 °C, reaction was stopped by adding 1 mL of ice-cold PBS. Cells were centrifuged as indicated above, and washed twice with ice-cold PBS. Cell pellets were resuspended in 0.2 mL of water and counted for radioactivity in UltimaGold XR liquid scintillation cocktail (Packard Instrument Co., Meridien CT, USA) [23]. Assays were run at least by triplicate. Cell viability was assessed by direct microscopic examination. Non-specific uptake and carry over were measured in transport mixture at without incubation (T_0_), or incubated at 4 °C.

### Plasmid construction and parasite transfection

TcAAAP069, TcBilbo1 and TcAQP genes (GeneDB IDs: Tc00.1047053504069.120, Tc00.1047053511127.20, and Tc00.1047053508257.140, respectively) were amplified using genomic *T. cruzi* DNA as template. Amplification products were subcloned into a modified pTREX expression plasmid called pTREXL [24] (TcAAAP069) and also fused to eGFP (TcAAAP069:eGFP and TcBilbo1:eGFP) or mCherry (TcAAAP069:mCherry and TcAQ.P:mCherry). Constructions were transfected into *T. cruzi* epimastigotes of Y and MJL strain as follows. 10^8^ parasites grown at 28 °C in LIT medium were harvested by centrifugation, washed with PBS, and resuspended in 0.35 mL of electroporation buffer (PBS containing 0.5 mM MgCl_2_ and 0.1 mM CaCl_2_). This cell suspension was mixed with 50 μg of plasmid DNA in 0.2 cm gap cuvettes (Bio-Rad Laboratories). The parasites were electroporated using a single pulse of (400 V, 500 μF) with a time constant of about 5 ms.

### Proline and ATP determinations

Proline determinations were performed using a ninhydrin colorimetric method as previously described [25]. ATP measurements were performed using the “ATP Bioluminiscence Assay Kit HS II” (Roche) according to the manufacturer instructions.

### Fluorescence Microscopy

Epimastigote samples from the days 1–7 after transfection were washed twice with PBS. After letting the cells settle for 30 min at room temperature onto poly-L-lysine coated coverslips, parasites were fixed at room temperature for 20 min with 4% formaldehyde in PBS, followed by a cold methanol treatment for 5 min. Slides were mounted using Vectashield with DAPI (Vector Laboratories). Cells were observed in an Olympus BX60 fluorescence microscope. Images were recorded with an Olympus XM10 camera.

### Western Blot analysis

Western Blots were performed using total *T. cruzi* extracts fractioned by electrophoresis in polyacrylamide denaturing gels and transferred to polyvinylidene fluoride (PVDF) membranes. The PVDF membranes were treated for 1 h with 5% non-fat dry milk in PBS and then incubated with the corresponding primary antibody overnight, (anti-triFlag diluted 1∶2500 –Abcam–; anti-arginine kinase diluted 1∶4000). Membranes were washed and incubated with the secondary antibody for two hours (anti-mouse HRP 1∶2500, Vector Labs). Detection was done by chemiluminescence (Pierce).

### Bioinformatics

Sequences from the Tritryps genome projects were obtained at TcruziDB (http://tcruzidb.org/) and GeneDB (http://www.genedb.org/). Analysis of the DNA sequence data were carried out using the software package Vector NTI v. 10.3.0 (Invitrogen) and the online version of BLAST at the NCBI (http://www.ncbi.nlm.nih.gov/). Local or online software were used under default parameters. The sequences were analyzed for the presence of transmembrane spans by using SOSUI v. 1.11 [26], TMHMM v. 2.0 [27], TMPRED v. 1.0 (http://www.ch.embnet.org/) and HMMTOP v. 2.0 (http://www.enzim.hu/). Topology was schematized using the TMRPres2D software (http://bioinformatics.biol.uoa.gr/). Phenogram representation of amino acid sequences was constructed using TreeView 1.6.6 (http://taxonomy.zoology.gla.ac.uk/).

### Statistics and kinetic parameters calculation

Each experiment was carried at least out three times (replicates). Data were analyzed as follows: first normal distribution was tested using a Kolmogorov-Smirnov test and all groups presented a normal distribution. Afterwards, groups were analyzed using a one-way ANOVA followed by a post-hoc Dunnet's multiple comparison test using a significance cut-off value of P<0.05. Standard procedures were used to determine kinetic parameters. K_m_ and V_max_ values were obtained by nonlinear regression fit of the data to the Michaelis-Menten equation. Statistics, curve fittings, V_max_ and K_m_ were calculated using the GraphPad Prism 6 software.

## Results

### Expression of the T. cruzi proline transporter in yeasts

**Figure 1:**
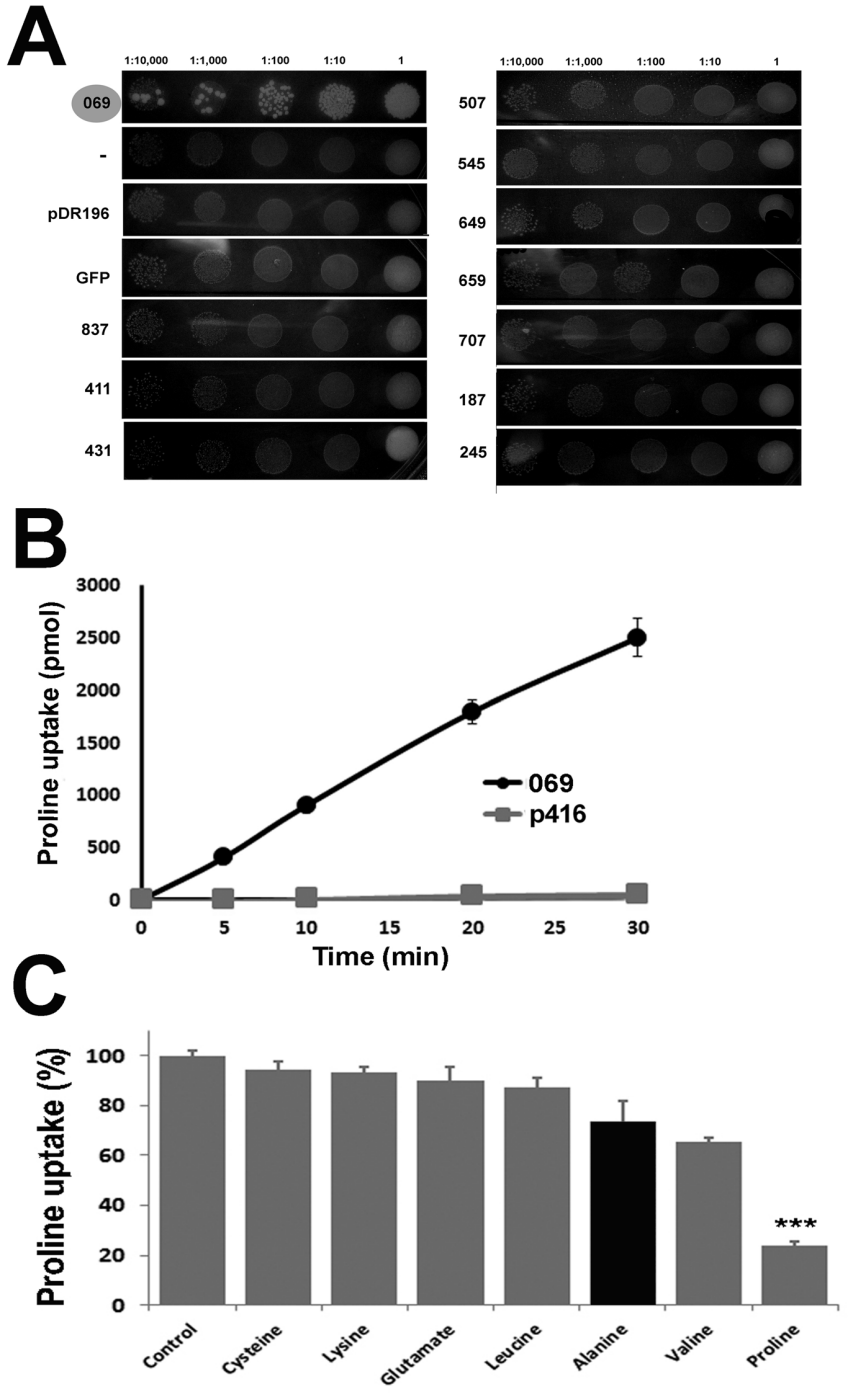
Expression and biochemical characterization of TcAAAP069 in yeasts. A) *S. cerevisiae* strain MG266 (gap1, put4) was transformed with the pDR196 plasmid harboring the genes: GFP, TcAAAP187, TcAAAP245, TcAAAP411, TcAAAP431, TcAAAP545, TcAAAP507, TcAAAP649, TcAAAP659, TcAAAP707, TcAAAP837 and TcAAAP069 or an empty pDR196 plasmid, and tested for growth in standard minimal medium (SD). Transformed strains were placed in drops containing approximately 10,000, 1,000 and 100 cells. Plates were photographed after 3 days of growth. B) L-(^3^H) proline uptake in yeasts transformed with the construct p416-TcAAAP069 (069, black line •) or an empty p416 control (p416, gray line 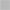). Transport experiments with 1 mM L-proline were performed using transformed yeasts grown up to exponential phase in SD medium an assayed for L- proline uptake in the interval 0–30 minutes. C) L-Proline transport inhibition assays were achieved using 1 mM L-proline and 10 mM of the different amino acid tested.

With the aim of identifying a *T. cruzi* proline transporter, ten genes from the previously characterized TcAAAP family have been subcloned in yeast expression vectors for complementation assays. After transformation of a yeast strain deficient in the proline permease (PUT4), cells were cultured in a medium containing proline as the solely nitrogen source. As figure 1A shows, only yeasts expressing the transporter TcAAAP069 were able to grow under these conditions. The identity of this transporter is in accordance with the data published by Inbar et al. [12].

To confirm the function of TcAAAP069, proline uptake assays were performed in the same yeast model (Figure 1B). The obtained transport rate was almost constant during 30 min. Calculated velocity for TcAAAP069 transformed yeasts was 85.9 pmol.min^−1^ (± 4.7) while control yeasts showed an inconsiderable activity (1.7 pmol.min^−1^, ±0.4). In addition, the estimated Michaelis-Menten constant (K_m_) for proline transport in yeasts transformed with TcAAAP069 was 11.64 mM (± 4.08).

In order to test the substrate specificity of TcAAAP069, proline transport assays were accomplished in the presence of different amino acids with dissimilar structures, in a 10-fold molar excess respect to the proline concentration. Interestingly, none of the tested amino acids produce a significant inhibition of proline uptake demonstrating the high-specificity of TcAAAP069. As a positive control an isotopic dilution using 10-fold higher proline concentration was included (Figure 1C).

### Functional and biochemical characterization of TcAAAP069 in T. cruzi

To further study the functional properties of the transporter, we constructed a *T. cruzi* epimastigote model that overexpresses TcAAAP069. These parasites provide a most appropriate physiological environment for additional experiments. Epimastigotes were transfected using the genes TcAAAP069, and also GFP as control subcloned in the *T. cruzi* expression vector pTREXL (pTREX-069 and pTREX-GFP). After the selection period, a Western Blot analysis was performed to verify TcAAAP069 expression, and a duplex band was observed (Supplementary Figure S1A, left panel); one corresponding to the permease and the other could be a fragment generated by a partial proteolysis or a post-translational modification.

**Figure 2:**
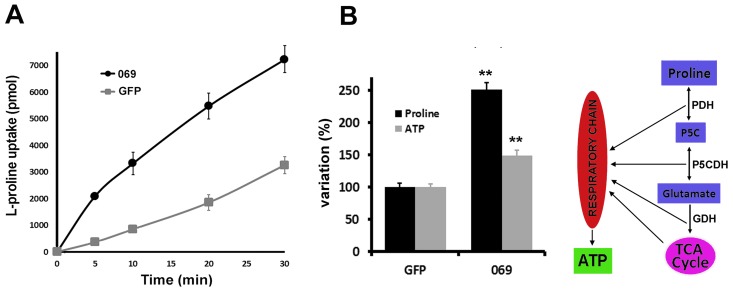
Functional studies of TcAAAP069 by overexpression in T. cruzi epimastigotes. A) Radiolabeled L-proline (1 mM) transport was measured in TcAAAP069 overexpressing parasites (069; black line •) and GFP controls (GFP; gray line 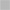) in the range 0–30 minutes (left panel) using 10^7^ parasites per assay. B) Intracellular proline (black bars) and ATP (gray bars) concentrations were measured in epimastigotes transfected with pTREX-TcAAAP069 (069) or the control pTREX-GFP (GFP). Intracellular proline and ATP concentrations were measured using the ninhydrin colorimetric technique and a bioluminescence method, respectively (left panel). Schematic representation of the different ATP-producing steps of the *T. cruzi* proline catabolism pathway (right panel).

Parasites transfected with TcAAAP069 showed an average proline transport rate of 417 pmol.min^−1^ (± 22) whereas in control parasites the transport rate was 73 pmol.min^−1^ (± 11) (Figure 2A).

Related to the parasites' growth kinetics, it was observed that epimastigotes overexpressing TcAAAP069 had a higher cell density than the GFP controls, about 30% between days 7 and 14 (Supplementary Figure S1A).

To confirm that TcAAAP069 is functional in other strain of *T. cruzi* from a different discrete typing unit (DTU) it was also expressed in the Y strain [16]. Expression was verified by Western Blot analysis and the same band pattern was obtained in samples from both strains (Supplementary Figure S1A, left panel). Additionally, proline transport was measured in the Y strain obtaining an increase in the transport rate of 35% respect to controls, in parasites overexpressing TcAAAP069 (Supplementary Figure S2A, right panel).

Given that one of the objectives of this work was to study the effects of the variation in the intracellular proline concentration, it was tested if an increase in the transport rate generates a higher proline concentration by accumulation of this amino acid. To address this point the total mass of intracellular proline was quantified in TcAAAP069 and GFP parasites. Proline concentrations were 6.4 mM (± 0.2) and 2.5 mM (± 0.1) for TcAAAP069 and GFP cells, respectively (Figure 2B, left panel). In accordance, parasites overexpressing TcAAAP069 from Y strain also showed an augmented proline concentration, from 1.9 mM (± 0.3) to 3.2 mM (± 0.7) (65%) (Supplementary Figure 2A, right panel).

On the other hand, proline can produce reduced metabolic intermediates prior and during the TCA cycle, and also acts as a direct electron donor to the respiratory chain (Figure 2B, right panel). Therefore, it could be expected that an increase in proline concentration alters the cell energy balance. To test this hypothesis ATP levels were determined in TcAAAP069 and GFP parasites showing an increase from 1.30 mM (± 0.1) to 1.92 mM (± 0.3) (48.3%) in epimastigotes overexpressing the proline permease (Figure 2B, left panel).

**Figure 3:**
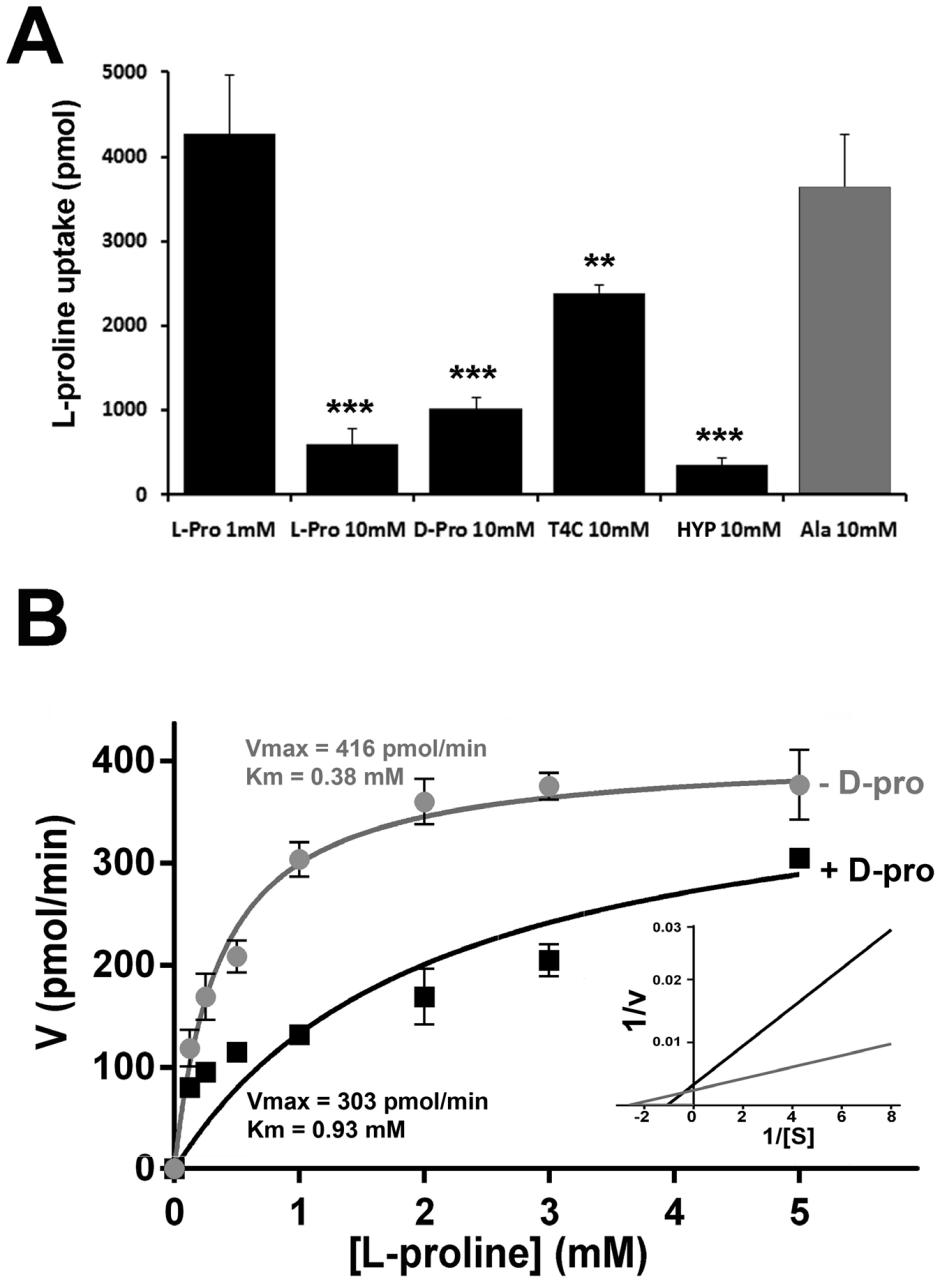
Transport inhibition assays. A) L- proline (1 mM) transport assays were performed in the presence of 10 mM of: L-proline (L-pro; 10-fold isotopic dilution), D-proline (D-pro), the proline analogue L-thiazolidine-4-carboxylic acid (T4C), the non-proteinogenic amino acid hydroxyproline (HYP) and alanine (Ala, gray bar). B) Kinetic analysis of L-proline uptake was performed using different concentrations of L-proline (from 0 to 5 mM) in the presence (+ D-Proline, black line •), or absence of D-proline 10 mM (- D-Proline, gray line 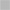). The Lineweaver-Burk plot is showed as an inset. Data were fitted using GraphPad Prism 6 software.

Previously reported inhibitors of proline transport and metabolism, such as L-thiazolidine-4-carboxylic acid (T4C) [13] and also the proline analogue hydroxyproline (HYP), were tested as TcAAAP069 inhibitors. Both compounds were assayed under the same conditions (10-fold molar excess). Although T4C produced a slight but significant inhibition of 44.21% (± 6.6), HYP treatment almost abolished proline transport producing a 91.77% (± 1.9) of inhibition (Figure 3A). These results are in accordance with the previously published by Magdaleno et al. [13].

Considering that amino acids competition assays performed in yeasts complemented with TcAAAP069 produced no significant inhibition on proline transport, an additional test was achieved using alanine, which was previously reported as a proline co-substrate in the *Leishmania* orthologue [12]. Unlike *Leishmania*, TcAAAP069 parasites did not show a significant inhibition on proline transport in the presence of 10-fold molar excess of alanine (Figure 3A).

Finally, given that there are very few cases of non-stereospecific amino acid transporters and particularly in trypanosomatids there is no previous report on this type of permeases, L-proline transport was assayed in the presence of D-proline. Surprisingly, D-proline inhibited L-proline transport by a 76.3% (±3.3) (Figure 3A). Kinetics studies of L-proline transport in the presence of D-proline (10 mM) showed an increase in the Michaelis-Menten constant (K_m_) from 0.38 mM (± 0.05) to 0.93 mM (± 0.48), whereas the maximum velocity (V_max_) remained constant (between 416.0 and 303.0 pmol.min^−1^; P value = 0.1745) adjusting to a competitive inhibition model (Figure 3B). The physiological role of D-proline transport could be associated to the presence of proline racemases in trypanosomatids, as discussed below.

### Bioinformatic analysis of TcAAAP069

All thirty-six members of the TcAAAP family, which were previously identified in the *T. cruzi* genome [14], have been schematized in a tree-type phenogram constructed according their amino acid identity (Supplementary Figure S3A). In the same branch of TcAAAP069 two additional genes were found. The first one (TcAAAP229) probably corresponds to an allele of TcAAAP069, which has a putative N-terminal extension of 62 amino acids length. Excluding this region, both alleles have 99% of amino acids consensus (Supplementary Figure S3B). The second protein of this branch, called TcAAAP733, has 42% of amino acid consensus with TcAAAP069. Both proteins have a very similar structure with 10 transmembrane spans and a very similar length of the intracellular N-termini, intra and extracellular loops (Supplementary Figure S3C). Considering that TcAAAP229 is probably an allele, TcAAAP733 could be a second proline transporter in accordance with the biochemical evidences that proposed the presence of two proline transport systems with different substrate affinities [2]. Nevertheless, yeasts complemented with TcAAAP733 showed no significant increase in L-proline transport but presented an active transport of an amino acid mix. However, TcAAAP733 substrates remain to be identified in a *T. cruzi* model thus it role as a proline transporter could be not ruled out.

### TcAAAP069 and resistance to ROS and trypanocidal drugs

**Figure 4:**
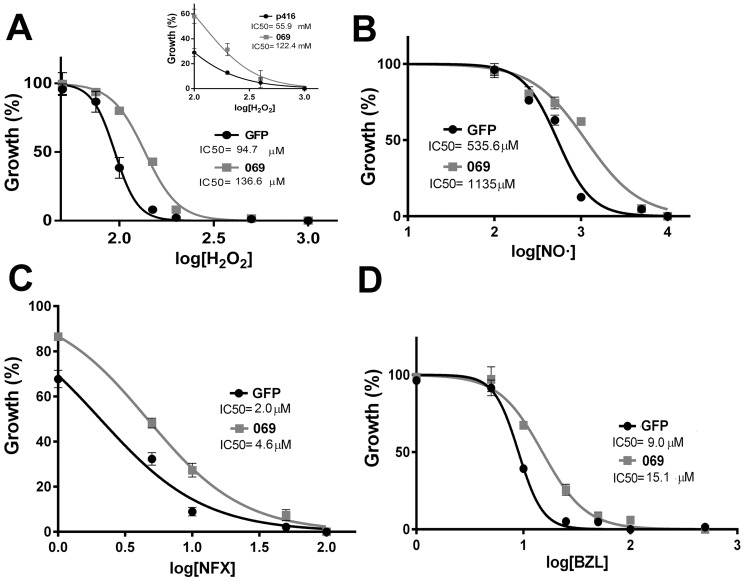
Effect of TcAAAP069 overexpression in stress and drug resistance. A) About 3×10^6^ parasites/ml (TcAAAP069 overexpressing parasites: 069, gray line 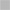; and GFP controls: GFP, black line •) were incubated with hydrogen peroxide at different concentrations from 0 to 1000 μM and counted 48 hours after treatment. IC_50_s were 136.6 μM and 94.7 μM for 069 and GFP, respectively. Yeast from the strain MG266 transformed with the plasmid p416-TcAAAP069 (069; gray line 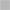) or an empty p416 control (p416; black line •), were grown in YPD medium. Aliquots of about 1 OD were subjected to a treatment with hydrogen peroxide at different concentration from 0 to 1000 mM and counted 48 h after treatment (inset). IC_50_s were 122.4 mM and 55.9 mM for 069 and p416, respectively. B) The same parasites' groups used at point A) were incubated with diethylamine NONOate (a nitric oxide donor) at different concentrations from 0 to 10 mM and counted 48 hours after treatment. IC_50_s were 1135 μM and 535.6 μM for 069 and GFP, respectively. C) The same parasites' groups used at point A) were incubated with nifurtimox at different concentrations from 0 to 100 μM and counted 48 hours after treatment. IC_50_s were 4.6 μM and 2 μM for 069 and GFP, respectively. D) The same parasites' groups used at point A) were incubated with benznidazole at different concentrations from 0 to 1000 μM and counted 48 hours after treatment. IC_50_s were 15.1 μM and 9 μM for 069 and GFP, respectively. All the curves were adjusted using GraphPad Prism 6 software.

In plants, it was proposed that proline itself could act as an intracellular ROS scavenger and also as a protection against different stresses by regulating enzymes of its own metabolism [28], [29]. However, the participation of proline transport systems in such processes has never been demonstrated. To test if an increased proline transport and intracellular concentration affect the cellular resistance to stress, treatments using different hydrogen peroxide concentrations were performed in yeasts expressing TcAAAP069. Results showed that TcAAAP069 significantly increases the yeasts resistance to hydrogen peroxide (IC_50_s 122.4 mM and 55.91 mM for TcAAAP069 and control, respectively. P value = 0.001) (Figure 4A, inset). Macrophages kill the trypomastigotes producing nitric oxide which is catalyzed by the inducible NO synthase [30]. Given these data, different stress conditions including hydrogen peroxide and diethylamine NONOate, a nitric oxide donor, were tested in TcAAAP069 and GFP parasites. Overexpression of TcAAAP069 produced a significant increase on the epimastigotes resistance to both, hydrogen peroxide and nitric oxide (IC_50_s: 136.6 μM and 94.7 μM H_2_O_2_; 1135 μM and 535.6 μM NO·, for TcAAAP069 and GFP cells, respectively. P values<0.0001) (Figures 4A and B). Accordingly, TcAAAP069 overexpression also increased resistance to hydrogen peroxide in *T. cruzi* Y strain parasites (IC_50_s: 146.2 μM and 123.6 μM H_2_O_2_; for TcAAAP069 and GFP cells, respectively. P value = 0.0003) (Supplementary Figure S1C).

Finally, the only trypanocidal drugs available for Chagas disease treatment at the present time, nifurtimox and benznidazole, were also assayed. Again, TcAAAP069 epimastigotes had a significant increased resistance (IC_50_s: 4.63 μM and 2.06 μM nifurtimox; 15.08 μM and 9 μM benznidazole, for TcAAAP069 and GFP cells, respectively. P values<0.0001) (Figures 4C and D). Together, these results suggest that proline itself is a fundamental cell defense under a wide variety of adverse conditions.

Since proline itself could trigger a cell stress response by increasing oxidative phosphorylation, the enzyme arginine kinase (E.C. 2.7.3.3) was used as a control of stress response proteins. Previous works [31], [32], [33] showed that arginine kinase expression increases during stress conditions, such as nutritional, pH and oxidative stresses. The enzyme expression was measured by Western Blot analysis using samples from TcAAAP069 and GFP parasites, and no differences were observed (Supplementary Figure 2C).

### Subcellular localization of TcAAAP069

**Figure 5:**
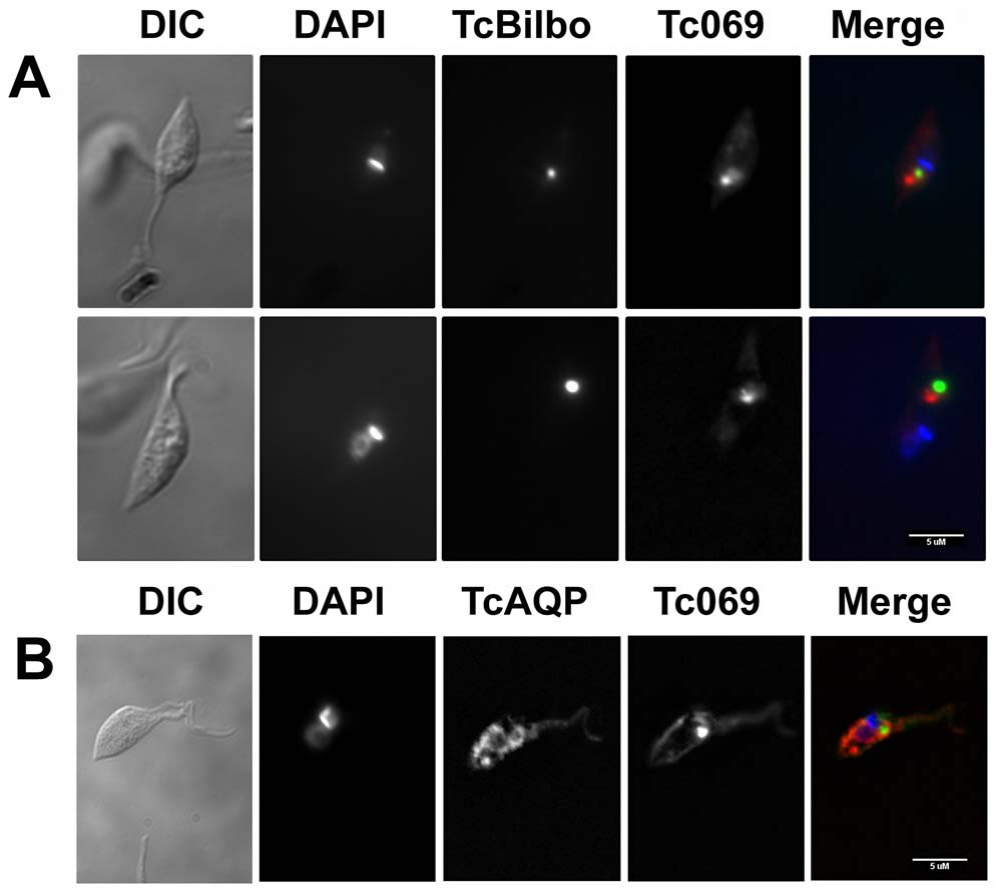
Subcellular localization of TcAAAP069. A) Parasites co-transfected with the pTREX-TcAAAP069:mCherry and pTREX-Bilbo1:eGFP were analyzed by fluorescence microscopy. Differential interference contrast (DIC), DAPI, TcBilbo (flagellar pocket marker), Tc069 and the merged image (Merge) are showed in the indicated columns. B) A similar analysis as in (A) was performed but using the acidocalcisomes and contractile vacuole marker aquaporin (TcAQP) instead of TcBilbo. Parasites co-transfected with the pTREX-TcAAAP069:eGFP and pTREX-TcAQP:mCherry were analyzed by fluorescence microscopy. White bars in the left corner of the merged images represent 5 μM.

Previous reports from our group indicate that other *T. cruzi* permeases from the TcAAAP family, such as the lysine and arginine permeases, and also putrescine-cadaverine transporter, are located, as well-defined structures, close to the flagellar pocket, probably associated to the contractile vacuole complex [16], [18], [34]. To determine whether TcAAAP069 has a similar localization to the mentioned permeases, genetics constructions containing the TcAAAP069 gene fused to GFP or mCherry were made. In addition, TcBilbo1 was fused to GFP and used as a marker of the flagellar pocket's collar, which demarcates a subdomain structure in the boundary of the flagellar pocket [16], [35]. After parasites transfection, TcAAAP069 fluorescence was mainly localized close to the flagellar pocket in a single focus but not colocalized with TcBilbo1 (Figure 5A). Similar results were obtained using the acidocalcisomes and contractile vacuole marker, aquaporin (TcAQP) fused to mCherry [36]; no colocalization between both proteins could be detected (Figure 5B). In addition, in a minor fraction of parasites TcAAAP069 was also located in the plasma membrane (Data not shown).

The fact that TcAAAP069 was found in the same cell region that the lysine, arginine and polyamine transporters suggests that probably all members of TcAAAP family could be concentrated in a unique region of interchange with the extracellular medium.

## Discussion

Along the life cycle, *Trypanosoma cruzi* must endure different extracellular conditions in the insect gut, the mammalian blood stream and also cell cytoplasm, presenting evolutionary adaptations to such environments [37], [38]. Among them, transport processes are rapid and efficient mechanisms, in comparison to biosynthetic routes, for supplying metabolites from extracellular media, and also to regulate the first step on many metabolic pathways. The proline transporter described in this work belongs to one of the major amino acid transporter families in trypanosomatids called TcAAAP [39], of which several members were already identified by our group [14]. This *T. cruzi* family of transporters comprises at least 36 genes coding for proteins with lengths of 400-500 amino acids and 10–12 predicted transmembrane α-helical spanners. One remarkable feature of these transporters is that they are absent in mammals [14].

In *T. cruzi*, two active proline transport systems were biochemically characterized [2]. All results herein presented, suggest that TcAAAP069 is the protein responsible for the transport systems described by Silber et al. However, considering the kinetics parameters (K_m_) TcAAAP069 is similar to system A, but its substrate specificity remains the system B [2]. These data open the possibility of a dual transport system, with different properties according the extracellular conditions, mediated by the same transporter.

Recently, a bi-specific proline-alanine transporter has been identified in *Leishmania donovani* (LdAAP24) which is the homologue gene of TcAAAP069 [12]. However, both transporters differs is some relevant biochemical properties, such as substrate specificity, and also the subcellular localization. While TcAAAP069 is located in a defined structure close to the flagellar pocket, LdAAP24 is uniformly distributed along the plasma membrane. It was also reported that LdAAP24 is the sole supplier, not only of intracellular proline, but also of alanine. Both amino acids play a critical role under osmotic stress conditions [12].

One interesting result herein presented, is the lack of sterospecificity of the proline transporter, D-proline was able to strongly decrease L-proline uptake with competitive inhibition kinetics. This feature represents a difference with the previous biochemical characterizations of proline transport systems, suggesting that TcAAAP069 is capable of transporting L and D-proline [2]. But some questions arise about the physiological role of a D-proline permease and the directionality of this transport. During infection of mammalian hosts, parasites secrete a proline racemase that contributes to parasite immune evasion [9]. *T. cruzi* proline racemases are encoded by two paralogous genes, one isoform is secreted and the other acts inside the cell [40]. One hypothesis is that extracellular L-proline is transported and “sequestered” by the parasites as D-proline to avoid being incorporated and consumed by host cells. Then, into the parasites' cytoplasm, D-proline is re-converted to its L- enantiomer and metabolized. Other possibility is that D-proline is incorporated in peptides or acts as an extra- or intracellular signal [11].

As previously mentioned, proline is a key metabolite involved in energy production and osmoregulation, however, its protective role under oxidative stress conditions was poorly studied. Previous evidences demonstrated that proline is a free radical scavenger in vitro [29]; it also modulates the intracellular redox environment and protects mammalian cells against oxidative stress [41]. In addition, a recent work showed that overexpression of *T. cruzi* proline dehydrogenase in a yeast model produces a decrease, not only in the intracellular proline concentration, but also in the resistance to hydrogen peroxide [5]. Here it was demonstrated that an increased proline concentration enhances protection against radical oxygen species generated not only by hydrogen peroxide but also by nitric oxide. Those are conditions to which the parasites are exposed permanently in its natural environment. In addition, TcAAAP069 transfected parasites presented an increased resistance to available trypanocidal drugs, nifurtimox and benznidazole, probably by affecting its mechanism of action involving oxidative stress. Interestingly, the *T. brucei* orthologue, TbAAT6 (GeneDB ID: Tb927.8.5450), besides proline, it is capable to transport the trypanocidal drug eflornithine (difluoromethyl ornithine) and mutations in this gene are sufficient to generate resistance [42]. These results could be relevant in terms of the effectiveness of treatments for Chagas disease, as the currently used drugs act, at least in part, by generating free radicals. Taken together, these data suggest that amino acid transporters may provide multiple, unexplored targets and gateways for therapeutic drugs.

## Supporting Information

Figure S1 **Expression of TcAAAP069 and parasites' growth kinetics.** A) Western blot analysis of TcAAAP069 expression was performed using two different *T. cruzi* strains overexpressing the TcAAAP069 gene: MJ Levin (DTU *T. cruzi* I) and Y (DTU *T. cruzi* II). Arrows on the left indicates the position of the 58 and 46 kDa molecular weight marker, and on the right, the position of the TcAAAP069 band duplex. B) Growth curves were calculated during 15 days from control (GFP; black line) and TcAAAP069 (069; grey line) MJ Levin strain epimastigotes.
(PDF)

Figure S2 **TcAAAP069 expression in Y strain and oxidative stress control.** A) Comparative proline transport (left panel) and proline concentration (right panel) were measured using GFP and TcAAAP069 transfected parasites. B) Oxidative stress assay using different concentrations of H_22_ Owas performed using GFP and TcAAAP069 transfected parasites. C) Arginine kinase (AK) expression was analysed by Western Blot as control for oxidative stress assays in samples from GFP and TcAAAP069 transfected parasites. As gel loading control was used the paraflagellar rod protein (PAR).
(PDF)

Supplemental Figure S3 **TcAAAP069 sequence analyzes.** A) Amino acid sequences of 36 members of the TcAAAP family were aligned using the ClustalW method and the phenogram representation was constructed using the TreeView 1.6.6 software. Numbers indicate the sequences groups defined according their amino acid identity. Dark grey box correspond to the proline transporter TcAAAP069 and the putative homologues. B) Putative proline transporters (TcAAAP733, TcAAAP229 and TcAAAP069) were aligned using the ClustalW method. Identical and equivalent residues were indicated as gray boxes. C) The sequences of TcAAAP069 (069) and TcAAAP733 (733) were analyzed for the presence of transmembrane spans using three different algorithms and topologies were schematized using the TMRPres2D software. The lower scheme represents a comparison of both transporters by overlapping their structures (overlap).
(PDF)
