# Supplementary material for: Proline Modulates the Trypanosoma cruzi Resistance to Reactive Oxygen Species and Drugs through a Novel D, L-Proline Transporter
Source: PLoS One. 2014 Mar 17;9(3):e92028. doi: 10.1371/journal.pone.0092028 (PMC3956872; doi:10.1371/journal.pone.0092028)
Supplement: Figure S1 — Expression of TcAAAP069 and parasites' growth kinetics. A) Western blot analysis of TcAAAP069 expression was performed using two different T. cruzi strains overexpressing the TcAAAP069 gene: MJ Levin (DTU T. cruzi I) and Y (DTU T. cruzi II). Arrows on the left indicates the position of the 58 and 46 kDa molecular weight marker, and on the right, the position of the TcAAAP069 band duplex. B) Growth curves were calculated during 15 days from control (GFP; black line) and TcAAAP069 (069; grey line) MJ Levin strain epimastigotes. (PDF) [file pone.0092028.s001.pdf]

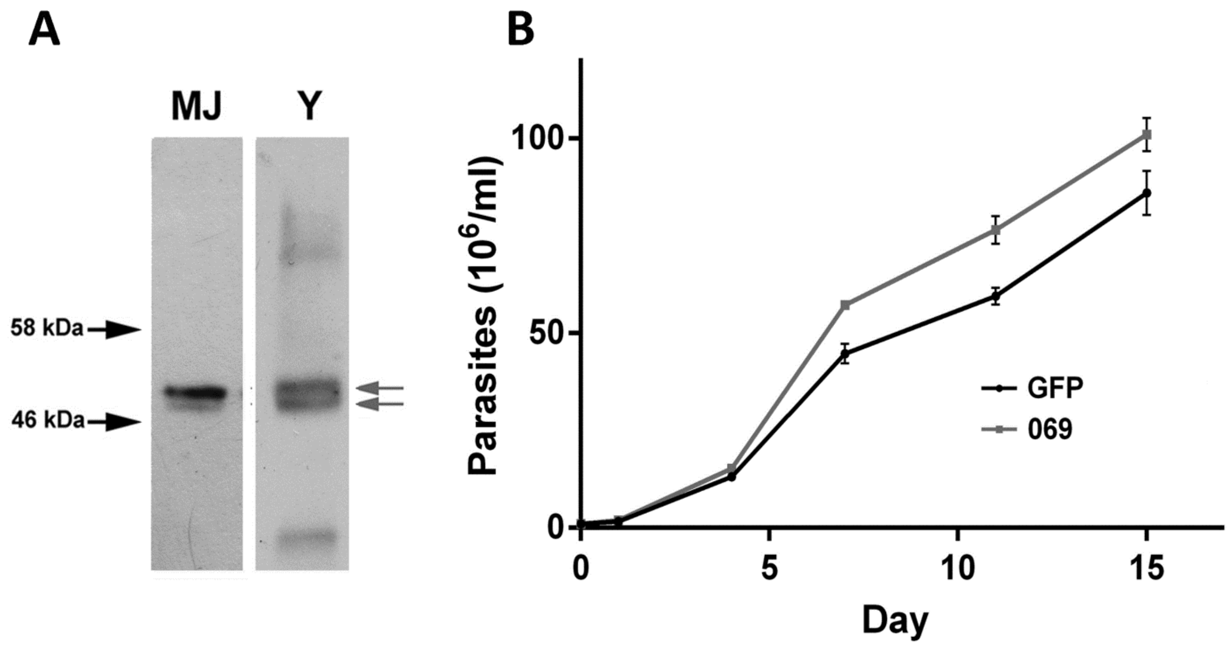

Supplemental Figure S1. Expression of TcAAAP069 and parasites' growth kinetics. A) Western blot analysis of TcAAAP069 expression was performed using two different *T. cruzi* strains overexpressing the TcAAAP069 gene: MJ Levin (DTU *T. cruzi* I) and Y (DTU *T. cruzi* II). Arrows on the left indicates the position of the 58 and 46 kDa molecular weight marker, and on the right, the position of the TcAAAP069 band duplex. B) Growth curves were calculated during 15 days from control (GFP; black line) and TcAAAP069 (069; grey line) MJ Levin strain epimastigotes.
