# Supplementary material for: Proline Modulates the Trypanosoma cruzi Resistance to Reactive Oxygen Species and Drugs through a Novel D, L-Proline Transporter
Source: PLoS One. 2014 Mar 17;9(3):e92028. doi: 10.1371/journal.pone.0092028 (PMC3956872; doi:10.1371/journal.pone.0092028)
Supplement: Figure S2 — TcAAAP069 expression in Y strain and oxidative stress control. A) Comparative proline transport (left panel) and proline concentration (right panel) were measured using GFP and TcAAAP069 transfected parasites. B) Oxidative stress assay using different concentrations of H22 Owas performed using GFP and TcAAAP069 transfected parasites. C) Arginine kinase (AK) expression was analysed by Western Blot as control for oxidative stress assays in samples from GFP and TcAAAP069 transfected parasites. As gel loading control was used the paraflagellar rod protein (PAR). (PDF) [file pone.0092028.s002.pdf]

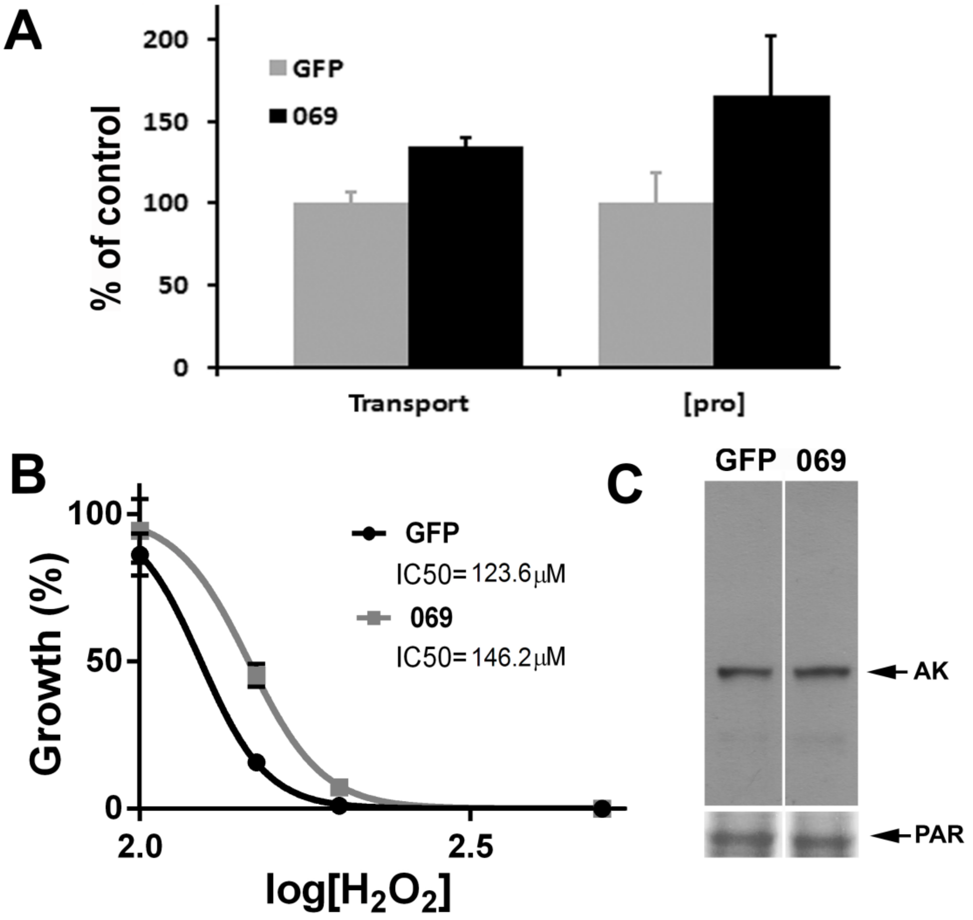

Supplemental Figure S2. TcAAAP069 expression in Y strain and oxidative stress control. A) Comparative proline transport (left panel) and proline concentration (right panel) were measured using GFP and TcAAAP069 transfected parasites. B) Oxidative stress assay using different concentrations of H_2O_2 was performed using GFP and TcAAAP069 transfected parasites. C) Arginine kinase (AK) expression was analysed by Western Blot as control for oxidative stress assays in samples from GFP and TcAAAP069 transfected parasites. As gel loading control was used the paraflagellar rod protein (PAR).
