# Supplementary material for: Proline Modulates the Trypanosoma cruzi Resistance to Reactive Oxygen Species and Drugs through a Novel D, L-Proline Transporter
Source: PLoS One. 2014 Mar 17;9(3):e92028. doi: 10.1371/journal.pone.0092028 (PMC3956872; doi:10.1371/journal.pone.0092028)
Supplement: Supplemental Figure S3 — TcAAAP069 sequence analyzes. A) Amino acid sequences of 36 members of the TcAAAP family were aligned using the ClustalW method and the phenogram representation was constructed using the TreeView 1.6.6 software. Numbers indicate the sequences groups defined according their amino acid identity. Dark grey box correspond to the proline transporter TcAAAP069 and the putative homologues. B) Putative proline transporters (TcAAAP733, TcAAAP229 and TcAAAP069) were aligned using the ClustalW method. Identical and equivalent residues were indicated as gray boxes. C) The sequences of TcAAAP069 (069) and TcAAAP733 (733) were analyzed for the presence of transmembrane spans using three different algorithms and topologies were schematized using the TMRPres2D software. The lower scheme represents a comparison of both transporters by overlapping their structures (overlap). (PDF) [file pone.0092028.s003.pdf]

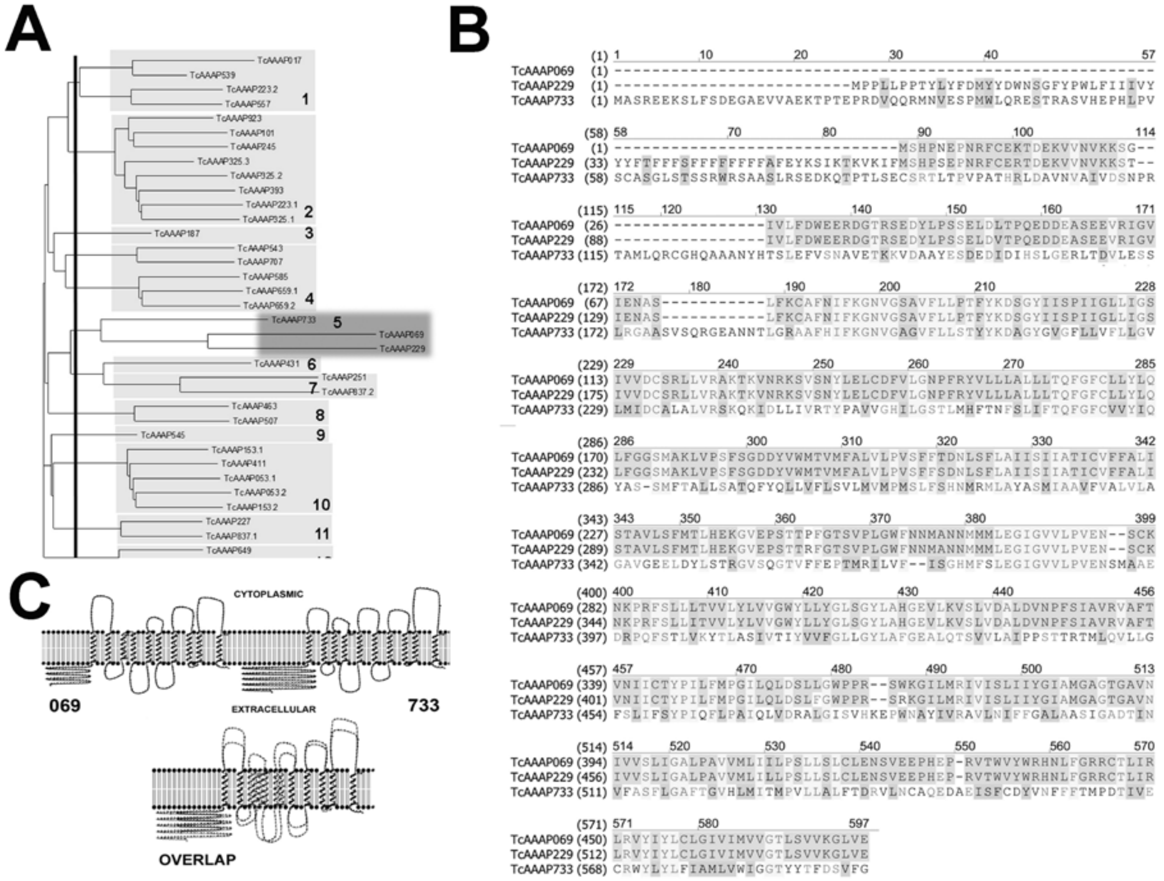

Supplemental Figure S3. TcAAAP069 sequence analyzes. A) Amino acid sequences of 36 members of the TcAAAP family were aligned using the ClustalW method and the phenogram representation was constructed using the TreeView 1.6.6 software. Numbers indicate the sequences groups defined according their amino acid identity. Dark grey box correspond to the proline transporter TcAAAP069 and the putative homologues. B) Putative proline transporters (TcAAAP733, TcAAAP229 and TcAAAP069) were aligned using the ClustalW method. Identical and equivalent residues were indicated as gray boxes. C) The sequences of TcAAAP069 (069) and TcAAAP733 (733) were analyzed for the presence of transmembrane spans using three different algorithms and topologies were schematized using the TMRPres2D software. The lower scheme represents a comparison of both transporters by overlapping their structures (overlap).
